# Acetabular Labral Tears in Patients with Sports Injury

**DOI:** 10.4055/cios.2009.1.4.230

**Published:** 2009-11-25

**Authors:** Chan Kang, Deuk-Soo Hwang, Soo-Min Cha

**Affiliations:** Department of Orthopedic Surgery, Chungnam National University School of Medicine, Daejeon, Korea.

**Keywords:** Actabular labral tear, Femoroacetabular impingement, Sports injury

## Abstract

**Background:**

We wanted to investigate acetabular labral tears and their correlation with femoroacetabular impingement in patients with sports injury.

**Methods:**

Among 111 patients who were diagnosed with the acetabular labral tears after arthroscopic treatment from January 2004 to December 2007, we selected 41 patients with sports injury. There were 12 cases of Taekwondo injury, 5 of golf injury, 4 of soccer injury, 3 of gymnastics injury, 2 of Hapkido injury, 2 of aerobics injury, 2 of rock-climbing injury, 2 of fitness training injury and 9 of other sports injuries. We checked the subtypes of acetabular labral tears and the accompanying femoroacetabular impingement. For the cases with accompanying femoroacetabular impingement, we investigated the subtypes according to the types of sports, gender and age. At last follow-up, we checked the Harris Hip Score (HHS), the Hip Outcome Score (HOS) sports scale and the percentage of patients who returned to their sports activity.

**Results:**

The average age of symptomatic onset was 26 years (range, 12 to 65 years). The ratio of males to females was 29 : 12. An average duration of the hip pain was 17 months (range, 1 to 60 months). The degenerative type of acetabular labral tears was the most prevalent with 32 cases (78%), and there were 9 cases (22%) of the partial tear type. Thirty cases (73%) were accompanied by femoroacetabular impingement. The average age of the 23 cases (56%) of the cam-type was 23 years (range, 12 to 48 years), and it was more likely to occur in men (87%) and for people practicing martial arts such as Taekwondo or Hapkido. An average age of the 5 cases (12%) of the pincer-type was 26 (range, 16 to 43 years), it usually occurred in women (60%) and for non-martial arts such as golf and gymnastics. There were 2 cases of the mixed type (cam + pincer-type). At 27 months follow-up, the HHS was 61 to 92 points, the HOS sports scale increased 43 to 75%, and the rate of returning to sports was 71%.

**Conclusions:**

In spite of the early expression of symptoms and the short duration of the acetabular labral tears, the high rate of degenerative acetabular labral tears in sports patients is likely associated with repetitive injury after the expression of symptoms. Femoroacetabular impingement in sports patients is seemed to be a cause of the early occurrence of acetabular labral tears. Because the possibility of acetabular labral tears is high in femoroacetabular impingement, sports patients may need to undergo early screening for the diagnosis and care of femoroacetabular impingement.

Acetabular labral tears (ALT) have been the subject of many studies regarding their pathophysiology, diagnosis and treatment, and their rehabilitation and prognosis.^1^-3) ALT has recently became diagnosable according to through history taking and specific physical examinations, and MR-arthrogram and 3D-CT are recognized as important diagnostic tools for most of the suspected cases of ALT and femoroacetabular impingement (FAI).^4^,5)

The advancement in the diagnostic methods and hip arthroscopy have allowed the lesions of the acetabular labrum that are caused by sports injuries to be diagnosed at an increasing rate and this has promoted discussion concerning the treatment.

In addition, ALT has been identified as a cause of hip pain in patients with FAI,^2^,^6^,7) and so ALT in athletic patients with FAI has received growing attention.^8^-14)

In this study, we investigated the different types of ALT in athletic patients. We explored the associations between ALT and FAI and we assessed the outcomes of arthroscopic treatment.

## METHODS

From January 2004 to December 2007 at our institution, ALT was the definite diagnosis for 111 patients who had undergone arthroscopic treatment under the provisional diagnosis of either ALT or ALT accompanied by FAI, and they were diagnosed using MR-arthrograms and 3D-CT. The patients had complained of acute or chronic pain and they had positive impingement test results. Of these 111 patients, 41 athletic patients were retrospectively reviewed for this study.

The mean age of the enrolled patients at the onset of symptoms was 26 years (range, 12 to 65 years). There were 29 males and 12 females. The mean duration of illness was 17 months (range, 1 to 60 months).

In this study, an athlete was defined as one who participated in a particular sport more than 4 hours a day and 5 days per week for more than 1 year until hip pain developed, and this was regardless of the patient having a professional or amateur status. Twelve patients were involved in Taekwondo, 5 in golf, 4 in soccer, 3 in gymnastics, 2 in Hapgido, 2 in aerobics, 2 in rock climbing, 2 in fitness training, 1 in baseball, 1 in swimming, 1 in inline skating, 1 in cycling, 1 in tennis, 1 in basketball, 1 in yoga, 1 in badminton and 1 in another athletic sport.

The patients who complained of hip pain underwent history taking, physical examinations, blood tests and radiography (the frog-leg lateral view, the false profile view and the cross-table lateral view) at the time of the visit.

The data obtained during history taking is as follows: the age at the time of the onset of hip pain, the age at the time of initial sports participation, the types of sports, the daily time spent on sports activities, the frequency of participation in sports in a week and the positions that aggravate the hip pain. For making the differential diagnosis, the patients were also asked about their past history of trauma and systemic diseases. The level of pain was rated according to the JOA pain scoring system set by the Japanese Orthopaedic Association: intermittent or continuous severe pain = 0 points, intermittent mild pain or occasional severe pain = 1 point, occasional mild pain = 2 points and no pain = 3 points.1)

On the physical examination, if passive flexion, adduction and internal rotation of the hip (impingement test)6) and if passive extension, abduction and external rotation of the hip (Patrick test)15) provoked snapping sounds or pain, then an anterior lesion of the acetabular labrum and a posterior lesion of the acetabular labrum was suspected, respectively.^8^,^9^,14)

Pain caused by systemic inflammatory disorders such as rheumatoid arthritis and ankylosing spondylitis was ruled out according to the diagnostic criteria.^16^,17)

For the patients with 0 or 1 point (the JOA pain score), a positive impingement test and suspected FAI based on the plain radiographs despite conservative treatment, an MR-arthrogram was performed for those patients with nonspecific plain radiographic findings, but with suspected ALT on the physical examination. 3D-CT was performed for those patients who met the above criteria and who had an obvious femoral head-neck bump and acetabular retroversion on the plain radiographs. The ALT observed on the MR-arthrogram was graded according to the classification of Czerny et al.18)

The ALT patients who presented with severe pain underwent arthroscopy for making the definite diagnosis and treatment. The patient was placed in the supine position under epidural anesthesia or general anesthesia. With the hip in 25° of abduction, traction was applied to the leg in the neutral position. In this position, around 8-10 mm of joint space widening was identified with a C-arm image intensifier. With the aid of a C-arm intensifier, a 15 gauge, long spinal needle was inserted at the anterolateral portal and 20-30 cc saline water was injected into the joint after the identification of the presence of air in the joint. A guide needle was inserted through the spinal needle and a 5 mm trocar was introduced into the joint. The trocar was replaced by the arthroscope, and the acetabulum and femoral head (central compartment) were observed through it. Anterior and posterolateral portals were additionally used, if necessary, for arthroscopic examination and treatment.11) The traction was then removed and an arthroscope was inserted with the hip in flexion to observe and treat the lateral acetabulum and femoral neck (the peripheral compartment).

The arthroscopically identified ALT was removed. In patients with ALT accompanied by FAI, a bump at the head-neck junction (the cam-type FAI) was removed by a burr (bumpectomy), acetabular retroversion (the pincer-type FAI) was treated with acetabuloplasty and the damage to the acetabular cartilage was done with chondroplasty.

According to the modified classification of ALT by Beck et al.,15) there are three types of ALT based on arthroscopic observation and the presence of degenerative tear, partial tear and complete tear. We studied and assessed the presence of FAI in the patients with ALT. According to the age, gender and type of sports, the FAI were subdivided into either the cam-type or pincer-type impingement. Taekwondo and Hapgido were categorized as martial arts and the rest were classified as non-martial arts. Any association between the FAI in the different sports activities and the ALT type was also assessed.

The Harris Hip Score (HHS), the Hip Outcome Score (HOS) the sports scale score and the percentage of patients returning to their preinjury sports were measured before and after the arthroscopic treatment, and during a mean of 27 months (range, 6 to 53 months) of the follow-up period and these values were compared to evaluate the treatment outcomes. All statistical analyses were done with using the Mann-Whitney U test, which is a non-parametric test. The *p*-values ≤ 0.05 were considered statistically significant.

## RESULTS

The most common type of tear was degenerative tear in 32 cases (78%). Partial tear was found in 9 cases (22%) and complete tear was not observed. Of the 41 patients, FAI accompanied the tear in 30 patients (73%): there were 23 cases (56%) of the cam-type FAI, 5 cases (12%) of the pincer-type FAI and 2 cases (5%) of the cam + pincer-type FAI (Table 1).

For the 23 patients with the cam-type FAI, the mean age at the onset of symptoms was 23 years (range, 12 to 48 years) and there were 20 (87%) males and 3 (13%) females. The cam-type FAI was found in 9 patients (64%) out of the 14 patients with injuries that were due to martial arts activities such as Taekwondo and Hapgido, and the cam-type FAI was found in 14 (52%) out of the 27 patients who were injured during non-martial arts activities. Therefore, the cam-type FAI appeared to be more common in those patients who were involved in martial arts activities (*p* = 0.023).

In the 5 pincer-type FAI patients, the mean age at the onset of symptoms was 26 years (range, 16 to 43 years) and there were 2 (40%) males and 3 (60%) females. Four (15%) of the 5 cases were found in patients who had been involved in non-martial arts activities such as golf and gymnastics and 1 (7%) was found in a martial arts athlete. Therefore, the pincer-type FAI seemed more prevalent in non-martial arts athletes (*p* = 0.041) (Table 2).

The prevalence of cam-type symptoms was higher in the martial arts athletes while the prevalence of pincer-type symptoms was higher in the non-martial arts athletes (Table 3).

During the 27 months of mean postoperative follow-up, the HHS increased from 61 points to 92 points and the HOS sports scale rose from 43% to 75%, showing a statistical significance (*p* = 0.032) (Table 4). Of the 41 patients, 29 (71%) recovered to the level of taking part in the sports they used to play before the appearance of symptoms: 7 (50%) of the 14 martial arts athletes and 22 (81%) of the 27 non-martial arts athletes took part in their old sports. Such a recovery rate was significantly high for the non-martial arts athletes (*p* = 0.033). The number of patients who recovered to play sports at a less intense level or they changed their type of sports activities was significantly high among the martial arts athletes (*p* = 0.018): 5 (36%) of the martial arts athletes and 4 (15%) of the non-martial arts athletes (*p* = 0.018). The number of patients who quit playing sports after surgery was larger among the martial arts athletes (*p* = 0.027) (Table 5): 2 (14%) of the martial arts athletes and 1 (4%) of the non-martial arts athletes.

## DISCUSSION

Since Altenberg19) first reported on acute and chronic pain in the hip caused by ALT in 1977, ALT has been the subject of study for many authors.^20^-22) The acetabular labrum deepens the acetabular socket and it prevents direct contact of the cartilage by being closely attached to the acetabular rim and maintaining the synovial pressure.^5^,23) ALT can be observed in patients with congenital disorders such as acetabular dysplasia, slipped capital femoral epiphysis, Legg-Calvé-Perthes disease, degenerative joint diseases, major trauma such as acetabular fracture and fracture of the neck of the femur, and continuous and repetitive minor trauma.24)

ALT frequently occur in relatively young patients in their 20s to 40s and it usually causes piercing pain deep in the inguinal area in the squatting position, when sitting and standing from a chair and when getting on and off a vehicle.6) According to DeAngelis and Busconi25) and Nicholls,14) the diagnostic specificity was high when their modified Thomas tests were positive, that is, when pain or clicking was elicited when the hip was extended from a fully flexed position in either internal or external rotation. The diagnostic specificity was high when logrolling of the extremity was positive in the study by Byrd et al.^2^,3) In the current study, all of the ALT patients had a positive impingement test.

Kim and Azuma26) observed the location of the peripheral sensory nerve when performing biopsy of the acetabular labrum and they described it as being engaged in nociceptive and proprioceptive functions. Their findings were congruent with those of Itoigawa et al.,27) and accordingly, the mechanism of hip pain in ALT could be explained. In a previous age-related morphologic and pathologic study of the acetabular labrum in adults, we also demonstrated the existence of a peripheral sensory nerve in the acetabular labrum in ALT patients with pain and there was a definite loss of symptoms following arthroscopic removal of the acetabular labrum.28)

There are many reports on ALT combined with FAI in athletes.^5^,^7^,^9^,^12^,^13^,^29^,30) Most of them were the cam-type FAI combined with anterosuperial ALT, and they were successfully treated with arthroscopy. In the current study, we also showed that an arthroscopic procedure, rather than a conservative one, resulted in a more satisfying HHS in the patients with ≥ IIA tears that were rated according to the classification of Czerny.^12^,20)

Therefore, we now better understand the mechanism of pain in ALT patients. Athletes who have FAI without pain can go through intermittent pain when a partial acetabular labral tear occurs due to repetitive stimuli, which also leads to a degenerative tear that causes constant pain.

To interrupt such a mechanism of pain, arthroscopic treatment of FAI is used, and FAI the main cause of ALT. This procedure attempts to prevent ALT by removing the bony protrusion (a femoral head-neck bump or acetabular retroversion) that causes ALT and repair the damage to the acetabular cartilage. However, considering that the efficacy of preventive treatment has not been fully examined, we believe that more studies that focus on its efficacy should be conducted. As of now, performing a screening test for asymptomatic FAI on young athletes and making sure they are aware of the risk of ALT appears to be the proper approach. In the current study, no patients underwent preventive treatment and ALT occurred in all of the patients. To treat this, we performed arthroscopic bumpectomy or acetabuloplasty, and we obtained remarkable outcomes.

We subdivided ALT into the degenerative, partial and complete tears.15) Degenerative tears are known to be common in the middle-aged and older populations, and they occur at a high rate in young athletes.^21^,^22^,28) We attribute this to the fact that the patients early hip pain was not well taken care of and repetitive stimuli was continuously applied at a high frequency on the lesion. Therefore, it seems reasonable to expect that degenerative tears affect not only the middle-aged and older people, but also young professional athletes.

Byrd et al.^2^,3) reported that the sports that cause ALT associated with FAI included ballet, football, hockey, baseball, golf and athletic sports and they showed that the intensity of sports and the extent of damage to the acetabular labrum were not correlated. In this current study, martial arts in which sudden shocks were applied on the rim when the hip was involved in more than the normal range of joint motion were more associated with ALT caused by the cam-type FAI rather than non-martial arts activities.

According to Philippon et al.,30) FAI is more prevalent in athletes than in non-athletes and continuous stimuli resulted in more ALT. In this current study, FAI was observed in 73% of the athletes with ALT. However, this should not be interpreted that FAI (a femoral head-neck bump and acetabular retroversion) is more common in athletes. Rather, it seems more plausible to think ALT were more prevalent in the athletes with FAI than in the non-athletes with FAI due to the stimuli applied during sports activities, and accordingly, FAI was more likely to be diagnosed in athletes. This can also be interpreted that in non-athletes with the same anatomical abnormalities, unless impingement occurs during daily activities, the acetabular labrum is not damaged and so symptoms cannot be felt.

In this current study, a statistical significance was found for improved HHS and HOS sports scale scores following arthroscopic treatment in all the cases. However, when the treatment results of martial arts players and non-martial arts players were compared, the former group was less likely to resume their preoperative sports and they were more likely to change or quit sports (Table 5). We attributed this to the patients' decisions based on the biomechanical characters of sports rather than the extent of damage to the acetabular labrum according to the type of FAI.

Conclusively, we believe that professional athletes and would-be professionals with hip pain should under go screening tests for ALT and FAI. In addition, we recommend that the patients diagnosed with ALT undergo arthroscopic examination and treatment for symptomatic improvement and resumption of their preoperative sports activities.

## Figures and Tables

**Table 1:**
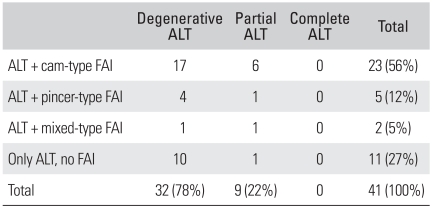
Types of Acetabular Labral Tears in the 41 Cases

ALT: Acetabular labral tears, FAI: Femoroacetabular impingement.

**Table 2:**
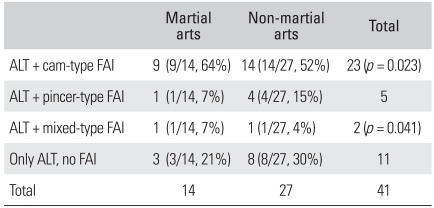
Relationship between the Sports Group and ALT

ALT: Acetabular labral tears, FAI: Femoroacetabular impingement.

**Table 3:**
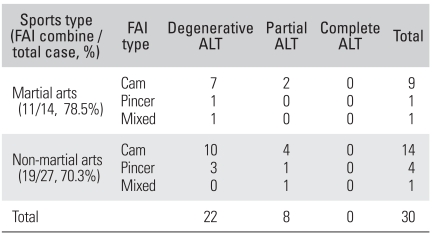
Relationship between the Sports Group and the FAI Type

FAI: Femoroacetabular impingement, ALT: Acetabular labral tears.

**Table 4:**
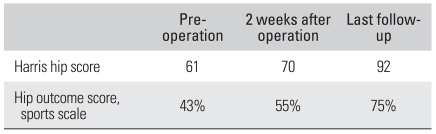
Results of Arthroscopic Treatment

**Table 5:**
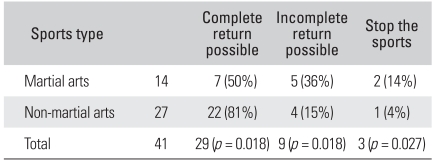
Recovery Rate for Returning to the Patient's Own Sports Activity
